# Coil Embolization of a Left Anterior Descending Coronary Artery-Pulmonary Artery Fistula: A Case Report

**DOI:** 10.7759/cureus.50521

**Published:** 2023-12-14

**Authors:** Shudipan Chakraborty, Kritika Luthra, Jay Shah, Moaaz Baghal, Ghulam Mujtaba Ghumman, Fnu Salman, Hemindermeet Singh, Syed S Ali

**Affiliations:** 1 Internal Medicine, Mercy Health - St. Vincent Medical Center, Toledo, USA; 2 Cardiovascular Disease, Mercy Health - St. Vincent Medical Center, Toledo, USA; 3 Cardiology, Mercy Health - St. Vincent Medical Center, Toledo, USA; 4 Interventional Cardiology, Mercy Health - St. Vincent Medical Center, Toledo, USA

**Keywords:** chronic stable angina, angina, coronary to pulmonary artery fistula, transarterial coil embolization, coronary artery fistula aneurysm, coronary artery

## Abstract

Coronary artery fistulas may be defined as abnormal connections between a coronary artery and either a heart chamber or the pulmonary artery. Although usually asymptomatic, they can become enlarged and rupture in rare instances, requiring prompt intervention. We present a case of a 66-year-old male patient with a left anterior descending-pulmonary artery fistula managed with coil embolization.

## Introduction

Coronary artery fistulas, the abnormal connections between a coronary artery and either a heart chamber or the pulmonary artery, can arise from congenital or acquired factors. Acquired causes include coronary angiography, pacemaker implantation, endomyocardial biopsy, and projectile injuries. While coronary artery fistulas are relatively uncommon, with an estimated prevalence of 0.17% among children and 0.64% among asymptomatic adolescents, they can pose risks. Typically asymptomatic, these fistulas may enlarge and, in rare instances, rupture, necessitating prompt intervention. This case report details a 66-year-old male patient with left anterior descending (LAD)-pulmonary artery fistula, successfully managed with coil embolization. Diagnosis often occurs incidentally during an echocardiogram and coronary angiography, with symptoms, when present, including syncope, congestive heart failure, recurrent angina, palpitations, and, rarely, sudden cardiac death. Recurrent angina and heart failure may be attributed to the coronary steal phenomenon or superimposed left ventricular volume overload from the fistula[1]. Multidimensional computed tomography angiography effectively reveals the morphology of coronary artery fistula. Treatment modalities for coronary artery fistulas include surgical ligation and transcatheter closure (TCC), a noninvasive alternative that avoids surgical complications. However, TCC may not be feasible in tortuous vessels or cases of large fistulas. Our case supports the notion that TCC of the coronary artery fistula is a reasonable alternative for eligible patients.

## Case presentation

A 66-year-old male patient, with a medical history of obstructive sleep apnea, essential hypertension, dyslipidemia, chronic kidney disease, and prediabetes, presented to the emergency department with typical substernal chest pain and shortness of breath. The chest pain was 8/10 in severity and radiated to the inner aspect of the forearm. The chest pain intensified with activity and subsided with rest. Additionally, the chest pain was associated with worsening shortness of breath. The shortness of breath was initially present on exertion but persisted even at rest during the Emergency Department (ED) evaluation.

Physical examination revealed bilateral pedal edema. Examination of the lung fields revealed decreased breath sound bilaterally in lower lung fields. The patient’s laboratory findings in the ED revealed an abnormal potassium level of 3.4 mmol/L, and an elevated pro B-type natriuretic peptide of 138 pg/mL, while the remaining laboratory findings were unremarkable. On arrival, the patient presented with hypertension, registering a blood pressure of 168/88 mmHg. The 12-lead electrocardiogram revealed sinus bradycardia with a heart rate of 50 bpm, first-degree atrioventricular (AV) block, and ST segment depression in V5 and V6. High-sensitivity troponins were negative on subsequent assessments. The patient reported that the last cardiac catheterization was 15 years ago, which was unremarkable. Home medication included omeprazole, gabapentin, albuterol inhaler, and bupropion.

**Figure 1:**
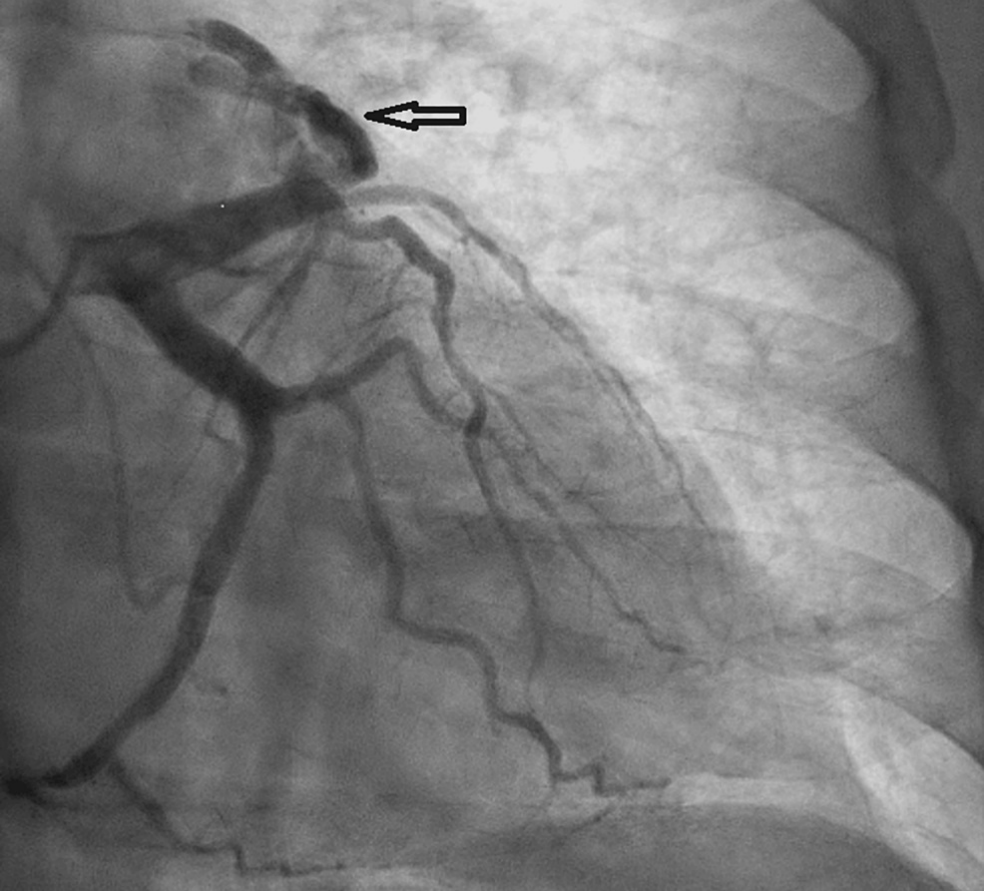
LAD to pulmonary artery fistula marked by the black arrow

**Figure 2:**
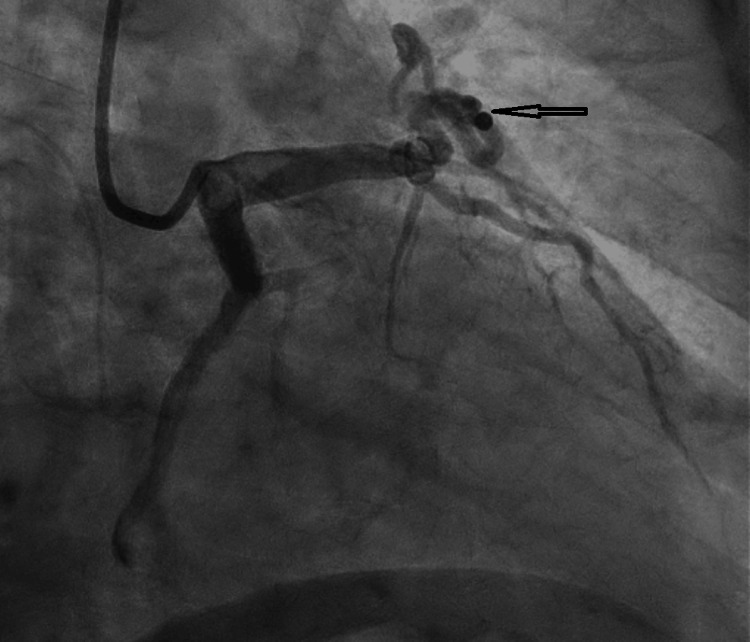
Status post coiling of the fistula between the left anterior descending and pulmonary artery

**Figure 3:**
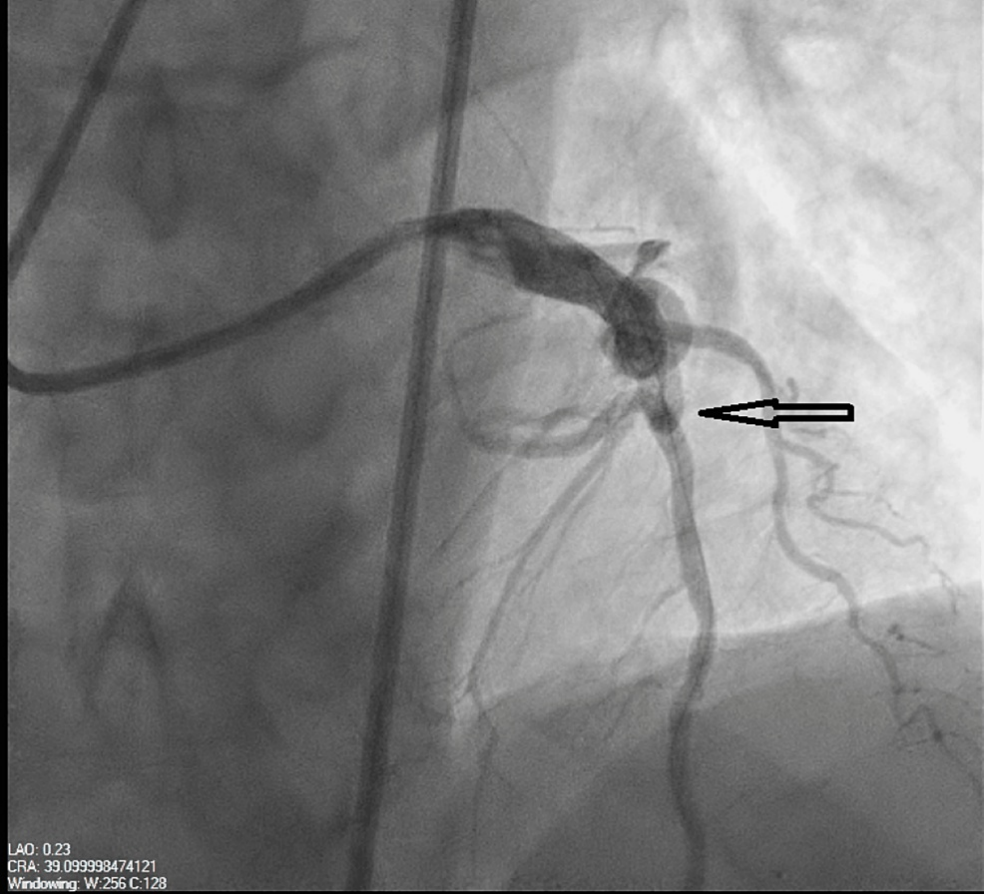
Status post stenting of the proximal left anterior descending

In response to concerns for unstable angina, urgent coronary angiography was performed, revealing 75% stenosis in the distal LAD, accompanied by a large arteriovenous fistula from the proximal LAD and draining into the pulmonary artery (Figure 1). Notably, the left main coronary artery was noted to be normal, while mild irregularities were observed in the left circumflex coronary artery. The mid-right coronary artery had 75% stenosis. Cardiothoracic surgery was then consulted, and they recommended coil embolization of the fistula, along with intervention on the significant coronary artery. Two weeks later, the patient underwent coil embolization of the AV fistula (Figure 2), along with percutaneous coronary intervention to address the LAD stenosis (Figure 3). Access to the substantial arteriovenous fistula connecting the proximal LAD to the pulmonary artery was achieved using a 0.014 wire, followed by a 5-French guide catheter. A 35-5-5 Mreye flipper coil was then deployed in the proximal portion of the fistula. The patient had an uneventful post-catheterization period and was discharged with a regimen comprising dual antiplatelet therapy, high-intensity statin, and ranolazine.

## Discussion

A coronary artery fistula occurs between a coronary artery and a pulmonary artery or one of the cardiac chambers. The clinical outcome of such a fistula depends on its termination site. If the site of termination is proximal to the tricuspid valve, it results in a left-to-right-sided shunt. If the site of the shunt is after the tricuspid valve, then left-sided volume overloading is the most likely consequence. The amount of blood shunting through the fistula depends on two key factors: the size of the fistula and the differences between systemic resistance and resistance in the terminating vessels/chamber. Notably, the right coronary artery is the most common site of origin for coronary artery fistulas, followed by the LAD, which is responsible for 30% of cases, and the left circumflex, which accounts for 18% of cases of coronary artery fistulas [2].

The clinical manifestation of coronary artery fistula varies from being completely asymptomatic, where the fistula is diagnosed accidentally via echocardiography and coronary angiography, to symptomatic presentations, such as syncopal episodes, congestive heart failure, anginal symptoms, palpitations, and, in rare instances, sudden cardiac death. The anginal episode and subsequent heart failure-like symptoms may be attributed to the coronary steal phenomenon that occurs due to the shunting of blood from coronary microcirculation toward the fistula termination site [3]. Complications associated with fistula include myocardial infarction, pulmonary hypertension, endocarditis, rhythm abnormalities, thrombosis, and rupture of the fistula.

Multi-dimensional computed tomography angiography (CTA) is very effective in revealing the morphology of coronary artery fistula, as it provides a three-dimensional visualization that facilitates a comprehensive delineation of the coronary artery anatomy. Studies have demonstrated that CTA detects coronary artery anomalies at a higher rate than traditional angiography. A CTA-based study reported the prevalence of coronary artery fistula to be 0.9%, which is higher than the known prevalence based on conventional angiographic findings (0.05-0.25) [4]. Coronary angiography is usually performed before surgery because it can provide a more detailed anatomy of the fistula.

The treatment modalities for coronary artery fistulas include surgical ligation and TCC. Notably, TCC is a noninvasive procedure that avoids all the complications of surgery. However, TCC cannot be performed in cases of a tortuous vessel or a large fistula[5]. The indications for surgical management of fistulas are severe pulmonary hypertension, prior history of bacterial endocarditis, asymptomatic fistulas with a left-to-right shunt of 30%, and fistulas with an enlarged aneurysm, particularly those with a diameter exceeding 3 cm, which poses a higher risk of rupture[6]. Other modalities for managing coronary artery fistulas include the use of covered stents, detachable balloons, and atrial septal defect devices [7]. Although no formal clinical studies are comparing TCC and surgical closure, TCC is gaining acceptance, especially for patients without underlying cardiac disease. In a study performed at the Mayo Clinic in Rochester involving 36 patients who underwent TCC, 89% (32) of the participants had no flow through the fistula after the procedure, whereas 11% (4) had very minimal flow[8].

## Conclusions

Our case report describes a successful coil embolization of a LAD to pulmonary artery fistula. The procedure was well-tolerated by the patient. Our case report adds to the existing extremely limited literature on the management of LAD to pulmonary artery fistulas. However, large-scale studies are necessary to formulate appropriate guidelines for the management of LAD to pulmonary artery fistulas and to determine the criteria for candidates who will require surgical ligation versus TCC.
